# Attitudes towards fibromyalgia: A survey of Canadian chiropractic, naturopathic, physical therapy and occupational therapy students

**DOI:** 10.1186/1472-6882-8-24

**Published:** 2008-05-31

**Authors:** Jason W Busse, Abhaya V Kulkarni, Parminder Badwall, Gordon H Guyatt, the Medically Unexplained Syndromes Study Group

**Affiliations:** 1 Department of Clinical Epidemiology and Biostatistics, McMaster University, Hamilton, Ontario, Canada; 2 Institute for Work & Health, Toronto, Ontario, Canada; 3 Division of Population Health Sciences, Hospital for Sick Children, Toronto, Ontario, Canada; 4 Canadian Memorial Chiropractic College, Toronto, Canada

## Abstract

**Background:**

The frequent use of chiropractic, naturopathic, and physical and occupational therapy by patients with fibromyalgia has been emphasized repeatedly, but little is known about the attitudes of these therapists towards this challenging condition.

**Methods:**

We administered a cross-sectional survey to 385 senior Canadian chiropractic, naturopathic, physical and occupational therapy students in their final year of studies, that inquired about attitudes towards the diagnosis and management of fibromyalgia.

**Results:**

336 students completed the survey (response rate 87%). While they disagreed about the etiology (primarily psychological 28%, physiological 23%, psychological and physiological 15%, unsure 34%), the majority (58%) reported that fibromyalgia was difficult to manage. Respondants were also conflicted in whether treatment should prioritize symptom relief (65%) or functional gains (85%), with the majority (58%) wanting to do both. The majority of respondents (57%) agreed that there was effective treatment for fibromyalgia and that they possessed the required clinical skills to manage patients (55%).

Chiropractic students were most skeptical in regards to fibromyalgia as a useful diagnostic entity, and most likely to endorse a psychological etiology. In our regression model, only training in naturopathic medicine (unstandardized regression coefficient = 0.33; 95% confidence interval = 0.11 to 0.56) and the belief that effective therapies existed (unstandardized regression coefficient = 0.42; 95% confidence interval = 0.30 to 0.54) were associated with greater confidence in managing patients with fibromyalgia.

**Conclusion:**

The majority of senior Canadian chiropractic, naturopathic, physical and occupational therapy students, and in particular those with naturopathic training, believe that effective treatment for fibromyalgia exists and that they possess the clinical skillset to effectively manage this disorder. The majority place high priority on both symptom relief and functional gains when treating fibromyalgia.

## Background

Fibromyalgia is a syndrome assigned to individuals presenting with chronic widespread pain, and who report of excessive tenderness at 11 of 18 specific muscle-tendon sites, for which no explanatory objective lesion can be found [1]. Approximately 2 percent of the general population in the United States suffers from fibromyalgia, with women affected 10 times more then men [2]. The cause of fibromyalgia remains an area of active debate and etiological theories implicate socio-demographic, physical and psychosocial factors [3,4], while some consider this syndrome the "medicalization of misery" [5]. Medical treatment directed towards fibromyalgia is highly variable and long-term prospective observational studies have found that patient outcomes, even in specialized rheumatology clinics, are typically poor [6,7]. Many North Americans, particularly those afflicted with conditions such as fibromyalgia that respond poorly to conventional medicine, use complementary and alternative medicine (CAM) [8,9]. Over 90% of patients with fibromyalgia use CAM and access CAM providers and allied health professionals [10-12], including chiropractors, physical therapists, occupational therapists, and naturopathic physicians [10-16].

While a number of studies of have established that allopathic physicians feel that patients with fibromyalgia are difficult to treat and that there is a general skepticism around the diagnosis itself [3,4,17-20], no formal studies have explored the attitudes of CAM providers and allied health professionals towards this challenging condition. A number of factors may condition CAM providers' and allied health professionals' attitudes toward patients with fibromyalgia and their behaviour toward these patients. These include belief in a biological versus a psychological etiology of fibromyalgia, optimism versus pessimism about its management; and optimism versus pessimism regarding their role in its management. Therefore, understanding how CAM providers and allied health professionals view fibromyalgia may provide insight into the clinical experiences of these patients and inform areas for education. The aim of the current study was to survey the attitudes of Canadian chiropractic, naturopathic, physical and occupational therapy students, in their final year of study, towards fibromyalgia; specifically, towards the etiology, diagnosis, and management of this syndrome.

## Methods

### Questionnaire development

With the assistance of epidemiologists and content experts, and reference to the previous literature, we developed a 13-item, English language questionnaire to examine chiropractic, naturopathic, physical and occupational therapy students' attitudes towards the etiology, diagnosis, and management of fibromyalgia. Recent graduates of each professional program of interest reviewed items to ensure that questions were relevant to course material being taught.

The final questionnaire framed the response options with 5-point Likert scales (strongly agree, agree, undecided, disagree, strongly disagree) as a previous report has shown that closed-ended questions result in fewer incomplete questionnaires than open-ended formats [21]. We also included an option for students to provide written comments regarding any other thoughts they may have on fibromyalgia.

We pretested the final questionnaire to a group of six recent graduates from the professional programs under study (3 physical therapists, 1 occupational therapist, 1 chiropractor, and 1 naturopathic physician) and one epidemiologist to evaluate if the questionnaire as a whole appeared to adequately measure attitudes towards fibromyalgia (face validity), and if the individual questions adequately reflected the domains of etiology, assessment, and treatment of fibromyalgia (content validity) [22]. The pretest participants also commented on the clarity and comprehensiveness of the questionnaire.

### Questionnaire administration

In January of 2005, through contact with the respective registrars, we identified all students in their final 4-months of study in the following programs: occupational therapy (n = 50) and physiotherapy (n = 50) at McMaster University, chiropractic (n = 150) at the Canadian Memorial Chiropractic College (CMCC), and naturopathic medicine (n = 135) at the Canadian College of Naturopathic Medicine (CCNM). CMCC is the only English-language chiropractic college in Canada, and CCNM is Canada's only accredited naturopathic college.

In April 2005 we administered, in person, surveys to each student group after a mandatory class; we collected surveys immediately after completion. Participants received a disclosure letter detailing the intent of the survey, and explicit instructions that they could choose not to complete the survey. Each institution's ethics review board approved the study.

### Statistical analysis

We generated frequencies for all collected data, and checked for difference in responses across items between professional groups with the chi-squared test. For the purpose of analysis, physical therapy and occupational therapy students were considered in the same group. One of us (JWB) grouped written comments according to themes to facilitate presentation. We determined *a priori *that certain attitudes may be correlated, and we examined statistical association between variables using the Pearson correlation coefficient. To address the issue of multiple comparisons we used the Bonferroni correction [23] to set our level of significance for our first model at 0.02 and at 0.01 for our second model.

#### Model 1

Variables associated with viewing fibromyalgia as predominantly a physical disorder: (patients with fibromyalgia have an as yet undiscovered physiological abnormality) and (exercise can be harmful to patients with fibromyalgia) and (patients with fibromyalgia typically demonstrate pathology on muscle tissue biopsy);

#### Model 2

Variables associated with viewing fibromyalgia as predominantly a psychological disorder: (symptoms of fibromyalgia are primarily caused by underlying mental illness) and (fibromyalgia is a useful diagnosis) and (fibromyalgia patients are difficult to manage) and (the role of a therapist is to minimize involvement) and (the role of a therapist is to refer for further assessment).

We hypothesized, *a priori*, that the following variables may be associated with respondent's belief that they possessed the clinical skills to effectively manage patients with fibromyalgia: (1) program of training, under the assumption that senior students in programs that focussed more on musculoskeletal complaints (physical and occupational therapy, and chiropractic) would be more confident than naturopathic students; (2) less confidence if they felt patients with fibromyalgia were difficult to manage; (3) belief in the etiology of fibromyalgia symptoms, with greater confidence in those who felt symptoms were more due to physical versus psychological factors; (4) greater confidence if they endorsed an active clinical role (provide symptoms relief, promote functional gains) versus not (minimize involvement, or refer to other clinicians); (5) less confidence associated with the belief that exercise was harmful; and (6) greater confidence associated with the belief that effective therapies had been established for fibromyalgia. These variables were all entered into a multivariable linear regression model [24].

To address the issue of multiple comparisons, we used the Bonferroni correction [23]. All comparisons were 2-tailed and a variable was considered statistically significant if it had a p-value < 0.01 in the final multivariable model. We report the unstandardized regression coefficient (b) and 95% confidence interval (CI) for each significant variable in the analysis. The regression coefficient represents the slope of the regression line – the amount of change in the y-axis due to a change of 1 unit on the x-axis. Each survey question is graded on a 5-point Likert scale and the value of 'b' represents the change in response score. We plotted residuals from the regression analyses to ensure that their distributions were reasonably normal. Multicollinearity was deemed concerning if the variance inflation factor for any independent variable was greater than 5 [25].

## Results

Respondents returned 336 of 385 surveys (response rate of 87%). The response rate for each professional group was as follows: physiotherapy and occupational therapy 61% (61 of 100), chiropractic 97% (142 of 150), and naturopathic medicine 99% (133 of 135); the difference in response rates was significant (p < 0.001). Of the respondents 42% were chiropractic students, 40% were naturopathic medicine students, and the remaining 18% were senior physiotherapy and occupational therapy students.

### General attitudes towards fibromyalgia

#### Etiology (Table 1)

**Table 1:** Response data to questions on the etiology of fibromyalgia

		**Response options (n) %**				
**Survey items**	**Student groups**	strongly disagree	disagree	uncertain	agree	strongly agree

Patients with FM have an as yet undiscovered physiological abnormality	PT/OT	(1) 2%	(12) 20%	(23) 38%	(21) 34%	(4) 7%
	DC	(2) 1%	(26) 18%	(64) 45%	(41) 29%	(9) 6%
	ND	(5) 4%	(18) 14%	(56) 42%	(47) 35%	(6) 5%
In most patients with FM, an underlying psychiatric illness is a prominent cause of their symptoms	PT/OT †	(5) 8%	(24) 39%	(19) 31%	(12) 20%	(1) 2%
	DC †		(26) 18%	(38) 27%	(70) 49%	(8) 6%
	ND	(6) 5%	(33) 25%	(40) 30%	(50) 38%	(2) 2%
Patients with FM typically demonstrate muscle tissue pathology on biopsy	PT/OT †	(1) 2%	(12) 20%	(23) 38%	(21) 34%	(4) 7%
	DC †	(2) 1%	(26) 18%	(64) 45%	(41) 29%	(9) 6%
	ND †	(5) 4%	(18) 14%	(56) 42%	(47) 35%	(6) 5%

† Significantly different reply when compared to other professions (chi-squared test, 2-sided, p ≤ 0.05)

FM = fibromyalgia

PT/OT = physiotherapy or occupational therapy

DC = chiropractic

ND = naturopathic medicine

Many respondents felt that psychiatric illness was a prominent cause of symptoms associated with fibromyalgia, with chiropractic students endorsing this belief most frequently (55%) and physical therapy and occupational therapy students expressing the greatest reserve (21%). Many respondents also agreed that patients with fibromyalgia typically demonstrate pathology on biopsy of muscle tissue, with physical and occupational therapy (41%) and naturopathic students (40%) endorsing this significantly more than chiropractic students (35%).

#### Assessment & diagnosis (Table 2)

**Table 2:** Response data to questions on assessment and diagnosis of fibromyalgia

		**Response options (n) %**				
**Survey items**	**Student groups**	strongly disagree	disagree	uncertain	agree	strongly agree

FM is a useful diagnosis	PT/OT †	(1) 2%	(3) 5%	(17) 28%	(35) 57%	(5) 8%
	DC †	(4) 3%	(49) 35%	(48) 34%	(37) 26%	(4) 3%
	ND	(3) 2%	(31) 23%	(35) 26%	(59) 44%	(5) 4%
Post-traumatic FM is a valid clinical diagnosis	PT/OT †	(1) 2%	(5) 8%	(47) 77%	(6) 10%	(2) 3%
	DC	(1) 1%	(19) 13%	(83) 59%	(39) 28%	
	ND †	(3) 2%	(10) 8%	(61) 46%	(53) 40%	(5) 4%
The tender point threshold (11 of 18), as defined by the ACR, provides useful information in the assessment of patients presenting with widespread musculoskeletal pain	PT/OT	(1) 2%	(17) 28%	(20) 33%	(21) 34%	(2) 3%
	DC †	(15) 11%	(42) 30%	(32) 23%	(49) 35%	(4) 3%
	ND †	(1) 1%	(24) 18%	(26) 20%	(73) 55%	(8) 6%
It is important to test for control points when performing tender point testing	PT/OT		(1) 2%	(17) 28%	(23) 38%	(20) 33%
	DC	(1) 1%	(5) 4%	(32) 23%	(67) 47%	(37) 26%
	ND †		(6) 5%	(35) 26%	(72) 54%	(18) 14%
A positive response on control point testing makes it far less likely that the person suffers from FM	PT/OT		(17) 28%	(34) 56%	(8) 13%	(2) 3%
	DC †	(6) 4%	(40) 28%	(61) 43%	(31) 22%	(3) 2%
	ND	(2) 2%	(40) 30%	(69) 52%	(15) 11%	(5) 4%

† Significantly different reply when compared to other professions (chi-squared test, 2-sided, p ≤ 0.05).

FM = fibromyalgia

ACR = American College of Rheumatology

PT/OT = physiotherapy or occupational therapy

DC = chiropractic

ND = naturopathic medicine

Less than a third of chiropractic students felt that fibromyalgia was a useful diagnosis, as compared to 48% of naturopathic students and 66% of physical therapy and occupational therapy students. While the majority of respondents felt it was important to test for control points when diagnosing fibromyalgia (71%), only 19% believed that a positive response on control point testing would reduce the likelihood of a diagnosis of fibromyalgia.

#### Treatment (Table 3)

**Table 3:** Response data to questions on treatment of fibromyalgia

**Domain & survey items**		**Response options (n) %**				
**Treatment – role**	**Student groups**	strongly disagree	disagree	uncertain	agree	strongly agree

My role in managing patients with FM is to provide symptom relief over functional gains	PT/OT †	(2) 3%	(13) 21%	(23) 38%	(15) 25%	(8) 13%
	DC	(1) 1%	(12) 9%	(34) 24%	(52) 37%	(43) 30%
	ND †	(1) 1%	(5) 4%	(25) 19%	(45) 34%	(56) 42%
My role in managing patients with FM is to promote functional gains over symptom relief	PT/OT †			(3) 5%	(12) 20%	(46) 75%
	DC †	(1) 1%	(7) 5%	(22) 16%	(56) 39%	(56) 39%
	ND			(17) 13%	(38) 29%	(76) 57%
My role in managing patients with FM is to minimize involvement as I have little to offer	PT/OT	(44) 72%	(14) 23%	(3) 5%		
	DC †	(61) 43%	(47) 33%	(25) 18%	(5) 4%	(3) 2%
	ND †	(111) 84%	(8) 6%	(7) 5%	(4) 3%	
My role in managing patients with FM is to refer for further investigations to identify cause	PT/OT †	(14) 23%	(16) 26%	(19) 31%	(11) 18%	(1) 2%
	DC †	(4) 3%	(16) 11%	(49) 35%	(47) 33%	(26) 18%
	ND †	(19) 14%	(34) 26%	(40) 31%	(18) 14%	(18) 14%

**Treatment – confidence**						

Patients with FM are difficult to manage	PT/OT †	(3) 5%	(5) 8%	(27) 44%	(24) 39%	(2) 3%
	DC †		(7) 5%	(33) 23%	(81) 57%	(21) 15%
	ND †	(1) 1%	(22) 17%	(43) 32%	(53) 40%	(14) 11%
Exercise can be harmful to patients with FM	PT/OT †	(25) 41%	(25) 41%	(3) 5%	(4) 7%	(4) 7%
	DC	(41) 29%	(74) 52%	(20) 14%	(7) 5%	
	ND †	(24) 18%	(84) 63%	(19) 14%	(5) 4%	
There are effective treatments for FM	PT/OT †		(1) 2%	(17) 28%	(42) 69%	(1) 2%
	DC †	(3) 2%	(30) 21%	(65) 46%	(43) 30%	(1) 1%
	ND †	(2) 2%	(9) 7%	(16) 12%	(71) 53%	(33) 25%
I am confident that I have the clinical skills to effectively manage patients diagnosed with FM	PT/OT		(12) 20%	(19) 31%	(28) 46%	(2) 3%
	DC †	(3) 2%	(29) 20%	(53) 37%	(53) 37%	(4) 3%
	ND †	(1) 1%	(9) 7%	(26) 20%	(62) 47%	(34) 26%

† Significantly different reply when compared to other professions (chi-squared test, 2-sided, p ≤ 0.05).

FM = fibromyalgia

PT/OT = physiotherapy or occupational therapy

DC = chiropractic

ND = naturopathic medicine

Most respondents felt that patients with fibromyalgia were difficult to manage, with chiropractic students (72%) endorsing this belief more often than naturopathic (50%) or occupational and physical therapy (43%) students. The respondents interpreted questions about the relative priority of symptom relief and functional gains in a different way than intended. The majority of chiropractic, naturopathic, physical and occupational therapy students (58%) provided internally contradictory answers: for one question they stated that symptom relief was more important then functional improvement and then endorsed the opposite priority in the next question. Overall, 65% endorsed symptom relief over functional gains and 85% functional gains.

Chiropractic students were least likely to agree that effective therapy existed (31%) or that they had the clinical skills required to manage patients with fibromyalgia (40%), whereas naturopathic students were most likely to endorse both of these items (79% and 73% respectively).

### Interaction of related beliefs

For our first model, the belief that patients with fibromyalgia have an as yet undiscovered physiological abnormality was weakly associated with the belief that exercise was harmful (r = 0.13; p = 0.02), but not with the expectation that muscle biopsy would show pathology (p = 0.3).

In our second model, the belief that the symptoms of fibromyalgia were largely the result of underlying mental illness was moderately associated with acknowledgement that this patient population was difficult to manage (r = 0.30; p < 0.01), and weakly with the belief that the role of CAM therapists was to minimize clinical involvement (r = 0.27; p < 0.01), and skepticism regarding the utility of fibromyalgia as a diagnosis (r = -0.18; p < 0.01). CAM therapists who indicated their clinical role with patients presenting with fibromyalgia was to refer to other therapists were also likely to endorse minimal clinical involvement as their role (r = 0.26; p < 0.01). Acknowledging that patient with fibromyalgia were difficult to manage was also moderately associated with endorsement of a minimal clinical role (r = 0.30; p < 0.01).

### Perceived ability to manage patients diagnosed with fibromyalgia

A number of variables were significantly associated with CAM student's perceived ability to manage patients with fibromyalgia; however, in our adjusted model only training in naturopathic medicine and greater belief that effective therapies existed for fibromyalgia remained significant (Table 4). Standardized residual plots showed no violation of model assumptions. The variance inflation factor was less than 2 for each independent variable, suggesting no issues with multicollinearity. Our model explained approximately 24% of the variance (adjusted R^2 ^= 0.24) in respondent's confidence in their skill set to appropriately manage fibromyalgia.

**Table 4:** Training and beliefs associated with confidence in managing fibromyalgia

**VARIABLE**	**Unstandardized regression coefficients from univariable analysis (95% CI)**	**p-value**	**Unstandardized regression coefficients from multivariable analysis (95% CI)**	**p-value**
Professional training:				
PT/OT	-0.20 (-0.46 to 0.06)	0.13		n/s
DC	-0.54 (-0.73 to -0.34)	<0.01		n/s
ND	0.68 (0.48 to 0.87)	<0.01	0.33 (0.11 to 0.56)	<0.01
FM patients are difficult to manage	-0.10 (-0.21 to 0.02)	0.11		n/s
FM patients have an underlying physiological abnormality	0.02 (-0.10 to 0.13)	0.79		n/s
Symptoms of FM are primarily due to mental illness	-0.06 (-0.17 to 0.05)	0.26		n/s
Clinical role in management of FM:				
Provide symptom relief	0.08 (-0.02 to 0.17)	0.14		n/s
Promote functional restoration	0.17 (0.05 to 0.29)	0.01		n/s
Minimize involvement	-0.27 (-0.39 to -0.16)	<0.01		n/s
Refer for further testing	-0.05 (-0.13 to 0.04)	0.25		n/s
Exercises can be harmful for patients with FM	0.02 (-0.10 to 0.14)	0.73		n/s
There are effective therapies for FM	0.48 (0.38 to 0.58)	<0.01	0.42 (0.30 to 0.54)	<0.01

95% CI = 95% confidence interval

n/s = not significant

FM = fibromyalgia

PT/OT = physiotherapy or occupational therapy

DC = chiropractic

ND = naturopathic medicine

### Written comments

Fifteen percent (9 of 61) of physical therapy and occupational therapy students provided a written comment, as did 20% of naturopathic medicine students (26 of 133), and 28% of chiropractic students (40 of 142). Physical therapy and occupational therapy students primarily focused on the need to approach treatment of fibromyalgia from a holistic perspective. The majority of written comments by chiropractic students' focused on the need for more research. The majority of naturopathic medicine student comments were split between these 2 themes. A number of chiropractic student's comments expressed concern over the potentially iatrogenic effect of labeling an individual with fibromyalgia. Table 5 presents the themes that emerged.

**Table 5:** Themes of written comment provided by respondents

	**Number of comments by student group**		
**Theme**	**PT/OT**	**DC**	**ND**

FM patients need to be educated to take control/responsibility for their recovery	1	4	2
FM patients can't tolerate the intensity of exercise supported in the literature	1		2
CAM has a large role to play in the treatment of FM*	1	2	5
I need more information on FM, or there needs to be more research on FM	1	10	7
FM patients need a mind-body/holistic/whole person approach to their assessment or rehabilitation (e.g. combinations of therapies such as exercise and cognitive behavioral therapy)	4	6	7
FM patients should be referred to multi-disciplinary pain programs	1	9	
Passive therapy breeds dependence in FM		2	
Labeling patients with FM can be harmful, as it can indoctrinate them into a disabling belief system		5	
It is critical to acknowledge FM patient's condition as they understand it, to emphasize that it's not 'all in their head'		1	2
I would not treat FM		1	
FM is part of multiple chemical sensitivity			1

* The PT/OT student emphasized nutrition, the DC student's emphasized spinal manipulative therapy, and the ND student's suggested homeopathic remedies, acupuncture, and nutrition

CAM = complementary and alternative medicine

FM = fibromyalgia

PT/OT = physiotherapy or occupational therapy

DC = chiropractic

ND = naturopathic medicine

## Discussion

### Summary of findings

Our survey of Canadian chiropractic, naturopathic, physical and occupational therapy students in their senior year found that the majority felt patients with fibromyalgia are difficult to manage. Most were unsure if an underlying physiological abnormality was responsible for symptoms or if muscle biopsy would demonstrate pathology, and many felt that underlying psychiatric illness contributed strongly to presentation.

The majority of respondents felt that fibromyalgia was a clinically useful diagnosis as was the American College of Rheumatology tender point threshold [1], but were uncertain as to the validity of post-traumatic fibromyalgia. Most students agreed it was important to test for control points when assessing for fibromyalgia, but were unwilling to abandon a diagnosis of fibromyalgia if control point testing was positive. Most students also agreed that effective therapies did exist for treatment of fibromyalgia, that exercise was not harmful, and that their clinical training allowed for effective management of fibromyalgia. Respondents endorsed both items suggesting that symptom relief should have priority over functional gains, and that functional gains should have priority over symptoms relief. We interpret this pattern of responses as demonstrating that most students consider both issues high priority.

Respondents who felt that fibromyalgia was the result of an undiscovered physiological abnormality were more likely to endorse exercise as harmful. Belief in a psychological etiology was associated with skepticism in regards to the utility of fibromyalgia as a diagnostic label, acknowledgement that this was a difficult patient population to manage, and a greater likelihood of endorsing minimal clinical involvement.

Professional training did influence responses to a number of survey items. Physical therapy and occupational therapy students were least likely to attribute symptoms of fibromyalgia to underlying psychiatric illness, were most likely to view fibromyalgia as a useful diagnosis, and were most likely to view their clinical role as promoting functional gains over symptom relief. Chiropractic students were most likely to ascribe symptoms of fibromyalgia to underlying psychiatric illness, least likely to endorse fibromyalgia as a useful diagnosis, and most likely to view their role in treatment to refer patients for further investigations. Chiropractic students were most likely to view fibromyalgia patients as difficult to manage, and least likely to endorse that there were effective therapies for this condition or that they had the clinical skills require to provide effective management. Naturopathic students were most likely view symptom relief as more important than functional gains, and were least likely to minimize their clinical involvement. Naturopathic students were also more likely to endorse that there were effective therapies for fibromyalgia and that they had the clinical skills required to provide effective management.

In our regression model, only training in naturopathic medicine and the belief that effective therapies existed for fibromyalgia were associated with greater confidence in managing patients with fibromyalgia. Most written comments by chiropractic, naturopathic, physical and occupational therapy students focused on the need for more research and education on fibromyalgia, and on the importance of managing this patient population from a holistic perspective.

### Strengths and limitations

The strengths of our study include a comprehensive sampling of Canadian chiropractic, naturopathic, physical and occupational therapy students in the final 4 months of their program of study, and a high survey response rate (87%) which limits nonresponder bias. Our results, however, may have limited generalizability to students outside of the groups we sampled.

### Relevant literature

As far as we are aware, there are no existing surveys of chiropractic, naturopathic, physical or occupational therapy students or practitioners that explore attitudes towards fibromyalgia. Studies have explored allopathic physicians' attitudes towards fibromyalgia and found that they typically find fibromyalgia difficult to treat and, similarly, these patients are more likely to be dissatisfied with treatment [17]. A qualitative study of 26 Swedish physicians reported they found patients with fibromyalgia to be demanding, aggressive in collecting information on their illness, illness focused, and medicalising [26]. Some authors have postulated that labels assigned to syndromes such as fibromyalgia are an artifact of medical specialization and have advocated for considering illness labels such as fibromyalgia, chronic fatigue syndrome, and irritable bowel syndrome, under the category of 'functional somatic syndromes' or 'medically unexplained syndromes' [27-29]. Diagnostic criteria for these syndromes frequently overlap, patients often meet the criteria for multiple syndromes, and similarities in patient characteristics, prognosis and response to treatment are common [27-30]. A recent survey of 1200 primary care physicians in Iowa (33% response rate) found that only 14% of respondents indicated very good or excellent satisfaction with managing patients with medically unexplained symptoms [18]. A study of 400 British general practitioners (75% response rate) found that less than half (44%) felt there were effective treatment options available with 93% reporting such patients were difficult to manage and 84% reporting their belief that personality problems predominated in this population [19]. The majority of senior chiropractic, naturopathic, physical and occupational therapy students who responded to our survey agreed in similar proportions that fibromyalgia was difficult to manage, but the majority expressed confidence that effective treatment options existed (57% in our sample), and in particular naturopathic students (79%).

Despite the uncertainly expressed by our respondents, fibromyalgia is not a muscle disorder and muscle biopsy is unremarkable aside from changes associated with deconditioning [31,32]. The prevalence of psychiatric illness among patients in whom fibromyalgia is diagnosed is high [33,34] and features of somatization predict the development of widespread pain [35], which the majority of our respondents acknowledged – particularly chiropractic students (55%).

The utility of tender point testing is debatable [36] and the American College of Rheumatology (ACR) criteria [1] were formulated as an aid to research rather than an assertion that the pathogenesis is understood [37]. Testing a single tender point appears to be similarly effective in distinguishing patients with fibromyalgia from controls [38]. Although control points were originally put forth to distinguish fibromyalgia from non-fibromyalgia pain, as the majority of patients labeled with fibromyalgia also report pain at control sites [31,37,39] it seems unlikely that this criteria is applied in practice, and our findings support this contention. Most of our respondents (57%) expressed uncertainty with regards to the validity of posttraumatic fibromyalgia as a diagnosis – although 44% of naturopathic students were supportive; however, in 1996 the Vancouver Fibromyalgia Consensus Group recommended that clinicians should "eliminate the terms 'reactive' and 'post-traumatic fibromyalgia"' due to a lack of any evidence to support such constructs [40]. Subsequent reviews of posttraumatic fibromyalgia [41,42], as well as a recent prospective observational study [43], have also failed to find compelling evidence in favor of a causal role of trauma in the onset of widespread bodily pain.

Trials have consistently established physical activation to be an effective therapy for fibromyalgia but often report high dropout rates due the initial increases in pain and stiffness immediately after exercise and patients' believing that exercise is harmful [44,45]. The struggle to provide both symptom relief and promote functional gains is not unique to our respondents, and underlies much of the challenge in implementing evidence-based treatment for fibromyalgia [46-48].

The majority of our respondents expressed confidence in their clinical skills to effectively manage fibromyalgia, although the available evidence is less clear. Sim and Adams [49] performed a systematic review of randomized controlled trials from 1980 to May 2000 of nonpharmacological interventions for fibromyalgia and concluded there was preliminary support of moderate benefit for aerobic exercise. In a systematic review by Ernst [50] regarding effectiveness of herbs and dietary supplements, the only agent with a possible benefit was capsaicin. Holdcraft and colleagues have conducted a systematic review of randomized and non-randomized controlled trials on CAM studies for fibromyalgia, up to 2002, to evaluate the empirical evidence for their effectiveness [51]. They found some evidence, although not conclusive, to support acupuncture, magnesium, S-adenosyl-L-methionine, and massage therapy. Goldenberg and colleagues reviewed the evidence for management of fibromyalgia, up to 2004, and concluded that, with regards to nonmedicinal therapies, strong evidence existed for cardiovascular exercise, cognitive behavioral therapy, and patient education [48].

## Conclusion

The high use of chiropractic, naturopathic, physical and occupational therapy by patients with fibromyalgia may be explained, in part, by the belief of senior students that effective treatments exist and in their confidence to effectively manage this challenging condition. Further research is required to establish if this confidence, particularly associated with naturopathic training, is justified. Belief that fibromyalgia is caused by an underlying physical lesion is associated with concern that exercise is harmful for this patient population; both beliefs could be detrimental to fibromyalgia patients. In addition, senior chiropractic, naturopathic, physical and occupational therapy students were conflicted over the validity of posttraumatic fibromyalgia and the role of control tender points in diagnosis, and between the desire to prioritize both symptom relief and functional gains when managing fibromyalgia. These areas should be considered targets for further education; specifically that the evidence to support physical trauma or an underlying physical lesion as a cause of fibromyalgia is poor, that exercise is not harmful, and that control points will be reported as tender by the majority of patients with fibromyalgia.

## Abbreviations

CAM: Complementary and alternative medicine; CMCC: Canadian Memorial Chiropractic College; CCNM: Canadian College of Naturopathic Medicine; ACR: American College of Rheumatology; FM: Fibromyalgia; PT/OT: Physiotherapy or occupational therapy student; DC: Chiropractic student; ND: Naturopathic medicine student

## Competing interests

The authors declare that they have no competing interests.

## Authors' contributions

JWB and GHG were involved in the study design and concept. JWB and PB conducted all data collection. JWB, AVK, and GHG conducted the analysis. All authors offered critical revisions to the manuscript; all approved the final version; JWB is the guarantor.

## Pre-publication history

The pre-publication history for this paper can be accessed here:


